# CBCT-Based Assessment of Mandibular Canal Position and Bone Morphology at Critical Osteotomy Sites for Sagittal Split Ramus Osteotomy

**DOI:** 10.3390/diagnostics16182916

**Published:** 2026-09-09

**Authors:** Mahzun Yıldız, Meyra Durmaz, Ömer Faruk Kocamaz, Mehmet Alp Eriş, Anıl Kamal, Merve Berika Kadıoğlu, Mehmet Emre Yurttutan

**Affiliations:** 1 Department of Oral and Maxillofacial Surgery, Faculty of Dentistry, Ankara University, 06560 Ankara, Türkiye; mzunyildiz22@gmail.com (M.Y.); mehmet.alp.eris@gmail.com (M.A.E.); anl.kamal37@gmail.com (A.K.); memreyurttutan@yahoo.com (M.E.Y.); 2 Department of Orthodontics, Faculty of Dentistry, Ankara University, 06560 Ankara, Türkiye; dtmeyradurmaz@gmail.com (M.D.); mkadioglu@ankara.edu.tr (M.B.K.)

**Keywords:** cone-beam computed tomography, inferior alveolar nerve, orthognathic surgery, sagittal split ramus osteotomy

## Abstract

**Background/Objectives:** Variation in the mandibular canal and surrounding bone may affect surgical risk during sagittal split ramus osteotomy (SSRO). This study examined whether canal position and bone morphology at SSRO-relevant sites differ according to mandibular sagittal position. **Methods:** This retrospective cone-beam computed tomography study included 72 adults classified as retrognathic, normal, or prognathic according to the SNB angle. Bilateral measurements from 144 mandibular canals were obtained at the mandibular foramen entrance, ramus–corpus transition, and mesial aspect of the second molar. Cortical and cancellous bone thicknesses, canal diameter, and canal-to-cortex distances were compared using one-way analysis of variance with Tukey’s post hoc test. Repeated measurements showed high reproducibility, with intraclass correlation coefficients exceeding 0.90. **Results:** At the mandibular foramen entrance, retrognathic patients had greater fusion of the buccal and lingual cortices-to-foramen and buccal cortex-to-canal distances than normal patients (both *p* < 0.05). Lingual cortical bone at the ramus–corpus transition was also thicker in the retrognathic group (*p* = 0.006). At the second molar level, the prognathic group showed greater buccal, lingual, and inferior cortical thicknesses but lower buccal cancellous bone thickness (all *p* < 0.05). Mandibular canal diameter did not differ significantly among the groups. **Conclusions:** Mandibular sagittal position was associated with regional differences in the bone surrounding the mandibular canal. Lower buccal cancellous bone thickness in prognathic patients may indicate less bony separation between the canal and the surgical field. These findings may inform patient-specific CBCT assessment and SSRO planning, although their relationship with surgical complications requires prospective clinical validation.

## 1. Introduction

Dentofacial deformities are structural abnormalities that impair the morphological and functional balance of the maxillomandibular complex and occur with varying frequencies in the general population [1,2]. These deformities not only result in aesthetic concerns but may also adversely affect essential functions such as mastication, speech, and respiration. Although orthodontic treatment alone may be sufficient in mild to moderate cases, advanced deformities often require orthognathic surgery aimed at three-dimensional repositioning of the jaws. Orthognathic surgery is a comprehensive treatment approach that involves repositioning and stabilizing skeletal segments following osteotomies in predetermined locations, thereby providing both functional and aesthetic improvements [3].

Sagittal split ramus osteotomy (SSRO) is one of the most commonly employed surgical techniques in the treatment of mandibular deformities. Owing to its ability to provide a broad bony contact surface, facilitate rigid internal fixation, and allow early postoperative function, it has become the preferred approach in contemporary orthognathic surgery. Nevertheless, the success of SSRO depends not only on the osteotomy design or fixation method but also on the anatomical structures encountered during surgery. In particular, the position of the inferior alveolar nerve (IAN) may directly influence surgical outcomes [4,5].

The most common complication associated with SSRO is neurosensory disturbance resulting from involvement of the inferior alveolar nerve. Although this condition is generally temporary, it may become permanent in some patients and negatively affect quality of life [6,7,8]. In addition, complications such as bad split, bleeding, infection, and fixation-related problems are also clinically significant. Among these, unfavorable splits may complicate surgical manipulation and adversely affect postoperative stability [9,10].

Although the development of these complications is multifactorial, the position of the mandibular canal and the relationship of the inferior alveolar nerve to the surrounding bony structures are considered key determinants. The proximity of the mandibular canal to the cortical plates and its distance from the osteotomy line may increase the risk of intraoperative nerve involvement [8,11,12]. In particular, a close relationship of the nerve to the buccal or lingual cortical plate, or its course within the osteotomy line, may increase the risk of both neurosensory complications and unfavorable splits [12,13]. Therefore, accurate three-dimensional evaluation of the inferior alveolar nerve may be beneficial during the surgical planning process.

Currently, three-dimensional imaging modalities such as Cone-Beam Computed Tomography (CBCT) allow detailed evaluation of the mandibular canal and may assist surgeons in identifying potentially risky anatomical variations prior to surgery [14,15]. Nevertheless, the findings in the literature regarding the relationship between mandibular canal position and complications following SSRO remain heterogeneous, and this association has not yet been fully clarified from a clinical perspective.

Previous CBCT-based studies have demonstrated that mandibular canal position and the amount of surrounding bone may vary according to skeletal pattern; however, the available evidence remains fragmented. Kalabalik et al. compared cortical and cancellous bone morphology between skeletal Class I and Class III individuals at molar levels, whereas Tseng et al. investigated buccal bone marrow distances among skeletal Classes I, II, and III [16,17]. More recently, de Castro et al. evaluated the positions of the mandibular foramen and mandibular canal across different skeletal classes using multiplanar reconstructions [18]. Nevertheless, previous studies have generally focused on selected mandibular regions, specific canal-to-cortex relationships, or intermaxillary sagittal classification. In the present study, the primary anatomical variable of interest was the sagittal position of the mandible itself relative to the cranial base. Therefore, the SNB angle was selected to characterize mandibular sagittal position rather than relying primarily on indices of maxillomandibular sagittal discrepancy. To our knowledge, the regional relationship between the mandibular canal and the separate cortical and cancellous bone compartments at multiple SSRO-relevant anatomical sites, including the FBLC region, remains incompletely characterized in relation to mandibular sagittal position.

Accordingly, the aim of this study was to evaluate the buccolingual position of the mandibular canal and the surrounding cortical and cancellous bone thicknesses at three anatomically and surgically relevant regions of the mandible—the mandibular foramen entrance region, the ramus–corpus transition region, and the mesial aspect of the second molar—and to investigate their relationship with mandibular sagittal position as determined by the SNB angle. In addition, differences in the distances between the mandibular canal and the surrounding cortical structures were assessed to characterize regional anatomical variations that may be relevant to individualized preoperative assessment for SSRO.

## 2. Materials and Methods

### 2.1. Study Design and Ethical Approval

This retrospective study was conducted through the evaluation of CBCT images archived in the Department of Oral and Maxillofacial Surgery, Faculty of Dentistry, Ankara University. Ethical approval for the study was obtained from the Ethics Committee of the same institution (Decision Date: 6 October 2025; Decision No: 14/20). The study was conducted in accordance with the ethical principles and standards outlined in the Declaration of Helsinki. Informed consent was obtained from all individuals prior to imaging, including consent for the anonymous use of their tomographic data for scientific research purposes.

### 2.2. Sample Selection

Individuals between the ages of 18 and 56 years who had completed skeletal growth and presented with mandibular second molars were included in the study. Patients with syndromic conditions, congenital or developmental anomalies affecting the mandible, or pathologies such as cysts or tumors were excluded. In addition, cases with missing mandibular second molars or radiographic artifacts were not included in the evaluation. The sample size was determined using G*Power 3.1 software (Heinrich Heine University, Düsseldorf, Germany). Based on a power analysis performed for one-way ANOVA, the effect size was set at f = 0.40 (medium-to-large), the significance level at α = 0.05, and the statistical power (1–β) at 80%. According to these parameters, a minimum of 66 individuals, including at least 22 participants per group, was required for the study. Considering the possibility of data loss, the sample size was increased by 10%, and a minimum of 24 individuals per group was targeted.

A total of 144 mandibular canals from 72 individuals were classified according to SNB angle values determined based on Steiner analysis. The SNB angle was measured using Dolphin Imaging software, version 11.8 (Dolphin Imaging & Management Solutions, Chatsworth, CA, USA). Based on previously reported SNB reference ranges, individuals with an SNB angle below 78° were classified as retrognathic, those with values between 78° and 82° as having a normal mandibular sagittal position, and those with an SNB angle above 82° as prognathic [19]. Based on this classification, the cortical and cancellous bone thicknesses surrounding the inferior alveolar nerve were analyzed.

### 2.3. Measurements

CBCT datasets were evaluated using multiplanar reconstruction in axial, sagittal, and cross-sectional views. The mandibular canal and the relevant cortical boundaries were identified by multiplanar navigation, and the measurement plane was adjusted according to the local anatomy at each predefined region. Measurements were then obtained on the section that most clearly demonstrated the mandibular canal and the corresponding cortical surfaces at the mandibular foramen entrance, ramus–corpus transition, and mesial aspect of the second molar. To evaluate the linear distances between the mandibular canal and the cortical bone, three anatomical regions considered clinically relevant for SSRO were predefined: the mandibular foramen entrance region, the ramus–corpus transition region, and the mesial aspect of the second molar.

**Figure 1:**
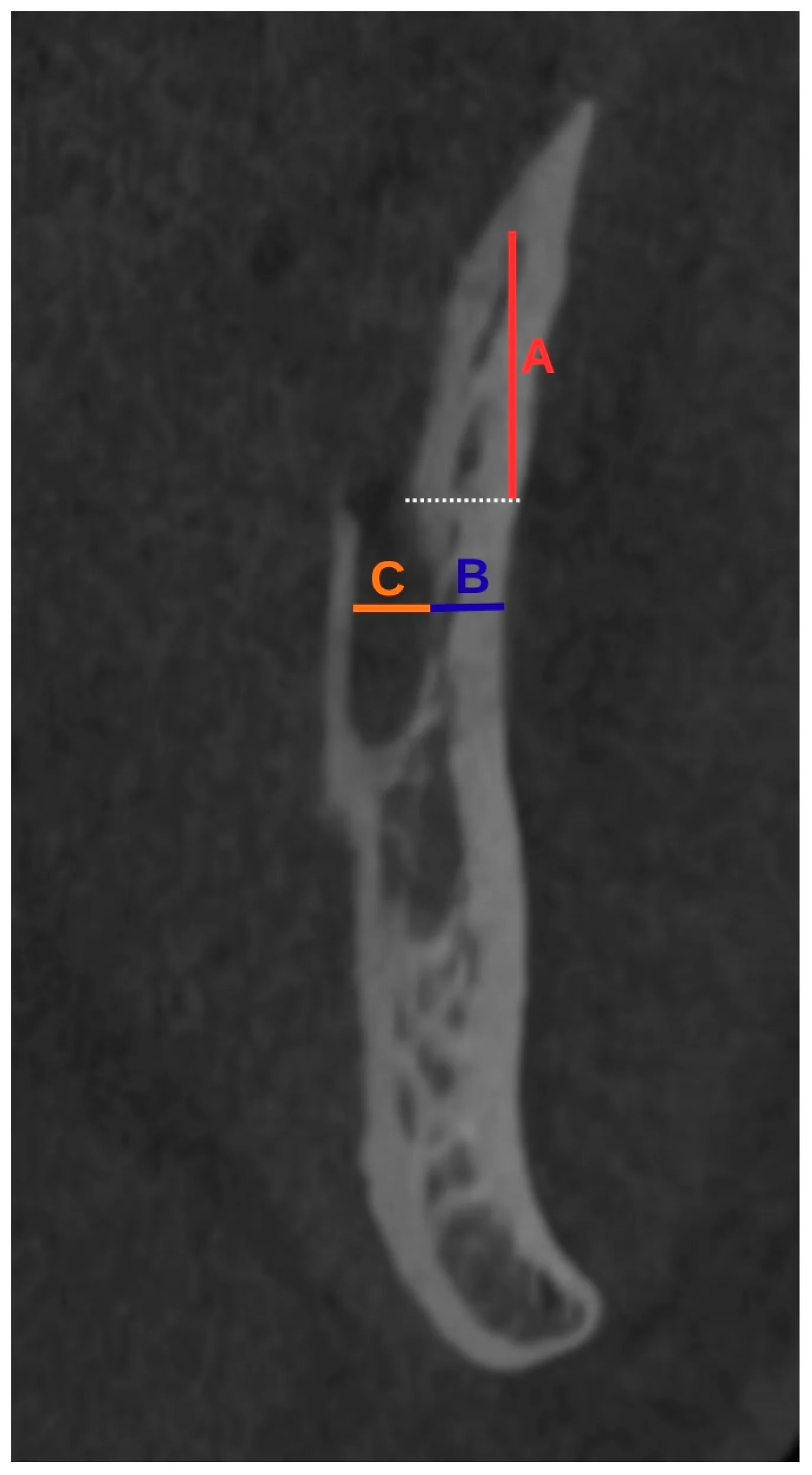
Sagittal sectional view of the inferior alveolar nerve at the mandibular foramen entrance region. The dashed line indicates the level of the mandibular foramen entrance. A: Distance between the fusion of the buccal and lingual cortices (FBLC) and the mandibular foramen; B: Distance between the buccal cortex and the mandibular canal; C: Diameter of the mandibular canal.

**Figure 2:**
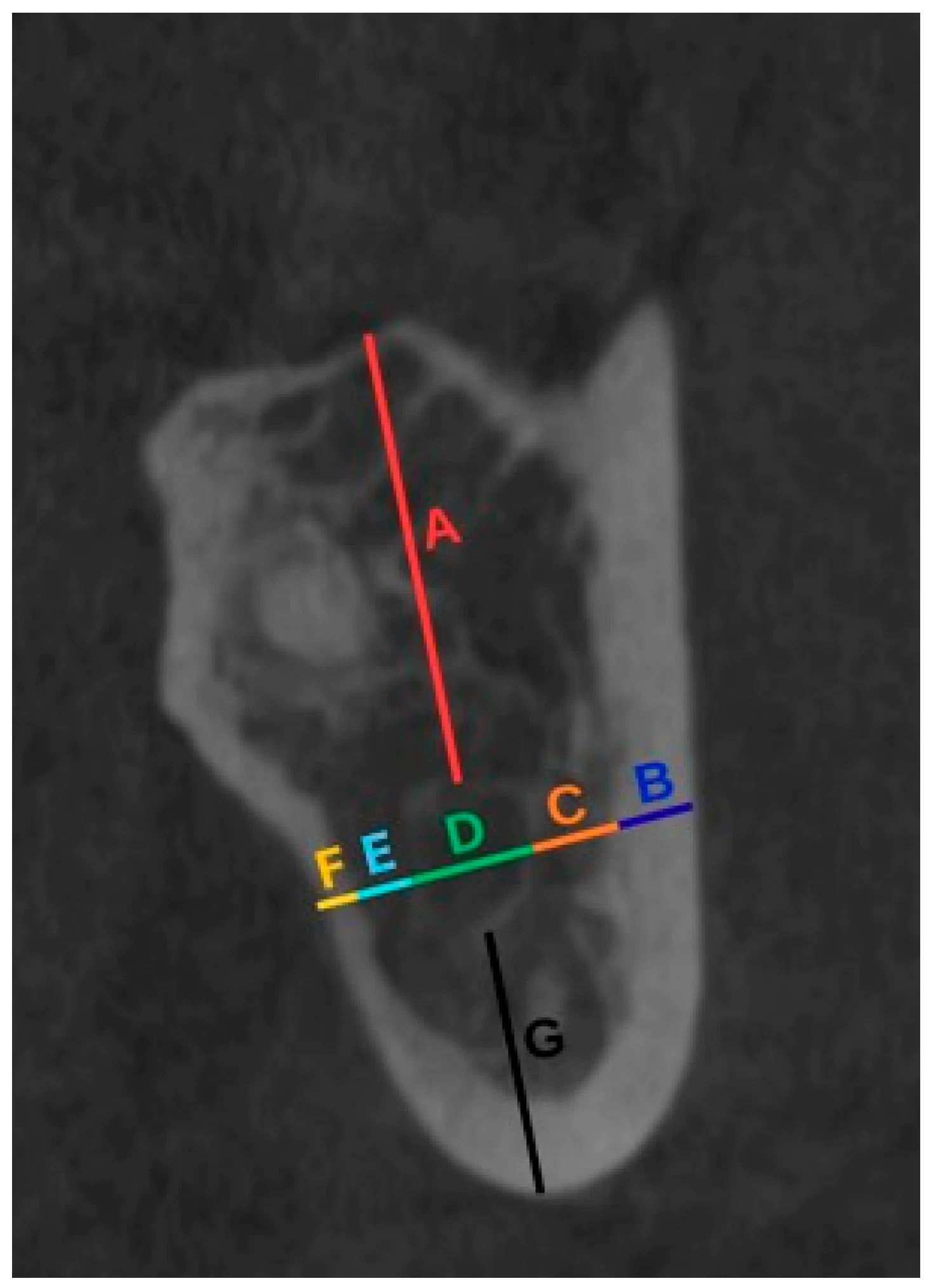
Sagittal sectional view of the transition region between the mandibular ramus and corpus. A: Distance between the mandibular canal and the alveolar crest; B: Buccal cortical bone thickness; C: Buccal cancellous bone thickness; D: Diameter of the mandibular canal; E: Lingual cancellous bone thickness; F: Lingual cortical bone thickness; G: Distance between the mandibular canal and the mandibular base.

**Figure 3:**
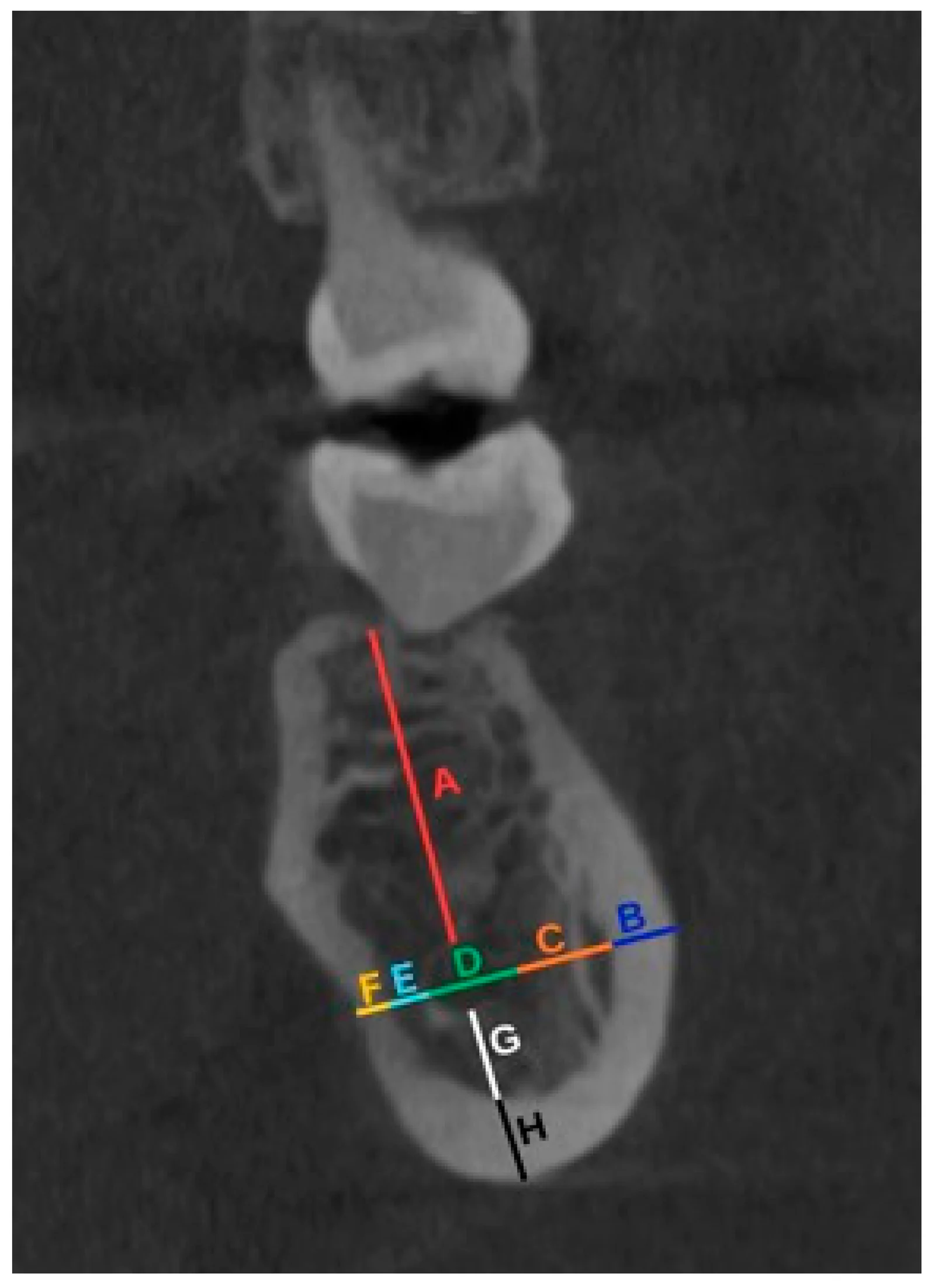
Sagittal sectional view of the inferior alveolar nerve at the mesial level of the second molar tooth. A: Distance between the mandibular canal and the alveolar crest; B: Buccal cortical bone thickness; C: Buccal cancellous bone thickness; D: Diameter of the mandibular canal; E: Lingual cancellous bone thickness; F: Lingual cortical bone thickness; G: Inferior cancellous bone thickness; H: Inferior cortical bone thickness.

The mandibular foramen entrance region was defined as the first image in which the mandibular foramen was visible on parasagittal sections. The ramus–corpus transition region was standardized separately for each side using multiplanar reconstruction. The anterior border of the mandibular ramus was identified by simultaneous navigation of the axial and sagittal views, and the most anterior point at the transition between the ramus and mandibular body was selected as the anatomical reference point. A cross-sectional measurement plane passing through this reference point and intersecting the mandibular canal was then generated by multiplanar reorientation. The same anatomical landmarking procedure was applied to both sides of all subjects. Thus, although the spatial orientation of the measurement plane varied according to individual mandibular morphology, the anatomical criterion used to define the ramus–corpus transition region remained consistent throughout the sample. In addition, the maximum diameter of the mandibular canal was measured in all three regions. In the mandibular foramen entrance region, the fusion between the buccal and lingual cortices (FBLC) was defined as the point where the buccal and lingual cortical plates merged on parasagittal CBCT images. In this region, the distance between the FBLC and the mandibular foramen, the distance between the buccal cortex and the mandibular canal, and the mandibular canal diameter were measured. The measurement points evaluated in the mandibular foramen entrance region are illustrated in Figure 1, those in the ramus–corpus transition region in Figure 2, and those at the mesial aspect of the second molar region in Figure 3.

### 2.4. Measurement Reliability and Reproducibility

All measurements were performed by a single experienced researcher. To assess measurement reliability, the same anatomical reference points on each individual’s CBCT images were measured twice at a four-week interval. The consistency between the measurements was evaluated using Intraclass Correlation Coefficient (ICC) analysis. ICC calculations were performed using a two-way absolute agreement model, and all 18 measurement parameters demonstrated ICC values greater than 0.90. These findings indicate a high level of measurement reproducibility and reliability.

### 2.5. Imaging Protocol

CBCT scans were obtained using a NewTom 7G cone-beam computed tomography device (Verona, Italy) with a 29 × 30 cm field of view (FOV). Radiation exposure parameters were automatically adjusted according to each patient’s body structure by the same technician. All imaging procedures were performed in accordance with the ALARA (As Low As Reasonably Achievable) principle.

The scans were performed with the patients in a standing position using head support devices to ensure stabilization, and routine radiation protection protocols applied in the clinic were followed throughout the imaging process. Images were obtained with a slice thickness of 1 mm and reconstructed with a voxel size of 0.600 mm. Following reconstruction, the acquired data were evaluated on axial, sagittal, and cross-sectional images using Synapse software (version 7.2.000; Fujifilm Corporation, Tokyo, Japan), and the required measurements were performed.

### 2.6. Statistical Analysis

Statistical analyses were performed using IBM SPSS Statistics version 26 (IBM Corp., Armonk, NY, USA). Continuous variables were expressed as the mean ± standard deviation, while categorical variables were presented as frequencies and percentages. Since the sample size in all groups exceeded 30, normality was assessed using skewness and kurtosis values, and all parameters were considered to show a normal distribution. Comparisons among the three groups (retrognathic, normal, and prognathic) were performed using one-way analysis of variance (ANOVA) for continuous variables. When statistically significant differences were detected, Tukey’s post hoc multiple comparison test was applied to identify the groups responsible for the differences. Categorical variables were compared using the chi-square test, and pairwise comparisons were performed with Bonferroni correction when significance was observed. All statistical tests were two-tailed, and a *p* value of <0.05 was considered statistically significant.

## 3. Results

Of the patients included in the study, 55.6% were female and 44.4% were male, indicating a relatively balanced sex distribution. The study groups were classified according to SNB angle, with equal numbers of individuals in the retrognathic (33.3%), normal (33.3%), and prognathic (33.3%) groups. Participant ages ranged from 18 to 56 years, with a mean age of 27.43 ± 6.39 years.

Comparison of sex distribution among the groups revealed a statistically significant difference (*p* = 0.004, *p* < 0.05). According to the post hoc multiple comparison analysis with Bonferroni correction, the proportion of male patients was significantly higher in the prognathic group compared with the retrognathic and normal groups.

The mean age was 25.79 ± 4.87 years in the retrognathic group, 31.21 ± 7.83 years in the normal group, and 25.29 ± 4.12 years in the prognathic group. A statistically significant difference in age was observed among the groups (*p* < 0.001). Further analyses performed following Bonferroni correction demonstrated that the mean age of patients in the normal group was significantly higher than that of both the retrognathic and prognathic groups.

Comparative findings regarding the demographic characteristics of the patients according to the study groups are summarized in Table 1.

When the measurements related to the mandibular nerve entrance region were compared among the study groups, statistically significant differences were observed for the distance between the FBLC and the mandibular foramen (*p* = 0.046) and the distance between the buccal cortex and the mandibular canal (*p* = 0.018) (*p* < 0.05). Further analyses demonstrated that both measurements were significantly greater in the retrognathic group compared with the normal group. In contrast, no statistically significant difference was found among the groups regarding mandibular canal diameter (*p* = 0.368, *p* > 0.05). The findings related to these comparisons are presented in Table 2.

Comparison of the measurements obtained from the ramus–corpus transition region revealed a statistically significant difference only in lingual cortical bone thickness (*p* = 0.006, *p* < 0.05). Further analyses demonstrated that patients in the retrognathic group had significantly greater lingual cortical bone thickness compared with both the normal and prognathic groups. In contrast, no statistically significant differences were observed among the groups regarding the distance between the mandibular canal and the alveolar crest (*p* = 0.294), buccal cortical bone thickness (*p* = 0.061), buccal cancellous bone thickness (*p* = 0.094), mandibular canal diameter (*p* = 0.95), lingual cancellous bone thickness (*p* = 0.616), or the distance between the mandibular canal and the mandibular base (*p* = 0.606) (*p* > 0.05). The mean and standard deviation values for these measurements are presented in Table 3.

Comparison of the measurements obtained from the mesial region of the second molar revealed statistically significant differences among the groups in terms of buccal cortical bone thickness (*p* = 0.035), buccal cancellous bone thickness (*p* = 0.032), lingual cancellous bone thickness (*p* = 0.024), lingual cortical bone thickness (*p* = 0.009), inferior cancellous bone thickness (*p* = 0.015), and inferior cortical bone thickness (*p* = 0.026).

According to Tukey’s multiple comparison analysis, buccal cortical bone thickness was significantly greater in the prognathic group compared with the retrognathic and normal groups, whereas buccal cancellous bone thickness was significantly lower in the prognathic group than in the retrognathic and normal groups. Lingual cancellous bone thickness was significantly greater in the retrognathic group compared with the normal group. In addition, lingual cortical bone thickness and inferior cortical bone thickness were significantly higher in the prognathic group than in the retrognathic and normal groups. Inferior cancellous bone thickness was also found to be significantly greater in the retrognathic group compared with the normal group.

In contrast, no statistically significant differences were observed among the groups regarding the distance between the mandibular canal and the alveolar crest (*p* = 0.211) or mandibular canal diameter (*p* = 0.645). The mean and standard deviation values for the related measurements are presented in Table 4.

## 4. Discussion

Patients with dentofacial deformities benefit substantially from SSRO not only in terms of aesthetic and functional improvement but also with respect to psychological and social well-being. Nevertheless, complications such as IAN injury and a bad split may adversely affect the overall success of this surgical procedure [16]. Several factors may contribute to the development of postoperative paresthesia, including surgery-related edema, pressure generated during screw fixation of the mandibular segments following osteotomy, and direct trauma to the IAN during vertical osteotomy or ramus splitting. In addition, patient age, the localization of the vertical osteotomy, and the extent of mandibular movement are among the other factors that may be associated with the development of postoperative neurosensory disturbances [12,17]. Therefore, the position of the inferior alveolar nerve and its relationship with the surrounding bony structures during SSRO should be carefully considered when evaluating potential surgical risks.

In the literature, classifications based on the ANB angle have frequently been used to evaluate sagittal skeletal patterns [17,20]. In the present study, retrognathic, normal, and prognathic groups were established based on the SNB angle, as it more directly reflects the sagittal position of the mandible relative to the cranial base. A significant difference in sex distribution was observed among the groups, with female patients being more prevalent in the retrognathic group and male patients in the prognathic group. Similarly, another study based on ANB angle classification reported a higher proportion of female patients in the Class II group and male patients in the Class III group [20].

In SSRO planning, knowledge of the localization of the FBLC, which represents the fusion point of the buccal and lingual cortical plates in the mandibular ramus, as well as the bone thickness in the osteotomy region, may help reduce the risk of complications. It has been reported that positioning the medial horizontal osteotomy at or above the level of the FBLC may increase the risk of unfavorable fractures [21]. Therefore, preoperative evaluation of the relationship between the FBLC and the mandibular foramen may be clinically useful in determining a safe osteotomy line. Scomparin and colleagues reported the mean distance between the FBLC and the mandibular foramen as 8.3 mm in males and 8.1 mm in females, with no significant difference between sexes [21]. Similarly, Noleto and colleagues, in a study comparing prognathic and retrognathic patients, reported the distance between the FBLC and the lingula as 8.95 mm in prognathic patients and 9.41 mm in retrognathic patients; however, these measurements were not evaluated according to sex [22]. In the present study, the FBLC–mandibular foramen distance and the buccal cortex–mandibular canal distance in the mandibular nerve entrance region showed significant differences among the groups. The greater values observed for both measurements in the retrognathic group may suggest the presence of a wider bony distance around the mandibular foramen in these patients, which may be relevant during preoperative planning of SSRO.

Kalabalık et al. compared mandibular corpus morphology in skeletal Class I and Class III individuals in terms of sagittal split osteotomy and reported that prognathic mandibles may be associated with an increased risk of neurovascular complications and bad splits [16]. Although no significant difference was observed among the groups in the ramus–corpus transition region in the present study, several measurements showed significant differences in the mesial region of the second molar, corresponding to the vertical anterior osteotomy line. In this region, buccal cortical bone, lingual cortical bone, and inferior cortical bone thicknesses were greater in the prognathic group compared with the retrognathic and/or normal groups. This finding may be related to the higher proportion of male patients in the prognathic group, as several studies have demonstrated that sex-related mandibular morphological differences may influence cortical bone thickness [21].

Previous studies have reported that positioning the vertical anterior osteotomy along the crest of the external oblique ridge, between the first and second molars, may represent a safer approach. Preference for this region has been associated with the presence of a greater bony distance buccal to the mandibular canal, potentially reducing the risk of IAN injury and bad split. Indeed, recent studies have also demonstrated that the location of the osteotomy line may influence both the split pattern and the risk of complications [23]. The findings of the present study also support this perspective. Although the total bony distance between the mandibular canal and the buccal external cortical surface of the mandible, defined as the sum of cortical and cancellous bone thicknesses, did not differ significantly among the groups, it demonstrated an increasing trend from the posterior toward the anterior region in all groups. This finding may suggest a more favorable anatomical relationship between the mandibular canal and the buccal cortical surface in the first and second molar region during planning of the vertical anterior osteotomy line.

The cancellous bone distance between the mandibular canal and the buccal cortical bone is an important anatomical parameter for preservation of the inferior alveolar nerve during SSRO. Yamamoto et al. reported that neurosensory disturbance was observed in all cases in which no marrow space was present between the mandibular canal and the buccal cortex, and that the risk of postoperative neurosensory complications increased significantly when this distance was 0.8 mm or less [12]. Similarly, Tseng et al. reported that buccal cancellous bone thickness varies according to skeletal pattern and that this distance is smaller in Class III patients compared with Class II patients [17]. Consistent with these findings, the significantly lower buccal cancellous bone thickness observed in prognathic patients in the present study compared with the retrognathic and normal groups may indicate closer anatomical proximity of the inferior alveolar nerve to the surgical field in this group. In contrast, the greater inferior cancellous bone thickness observed in the retrognathic group may provide a wider bony buffer zone around the inferior alveolar nerve during the osteotomy performed at the mandibular base region in SSRO.

In the present study, mandibular canal diameter showed a decreasing trend from the posterior toward the anterior region. This finding suggests that the morphological characteristics of the mandibular canal may vary regionally along its course. Likewise, a recent CBCT study reported that mandibular canal morphology and anatomical variations may differ among individuals and across different mandibular regions [24].

The present study has several methodological strengths, including the use of a standardized multiplanar measurement protocol, repeated measurements by a single examiner, and an a priori sample size calculation. The high intraobserver ICC values indicate good measurement reproducibility; however, reproducibility should not be interpreted as evidence that potential limitations related to image resolution or measurement methodology are absent.

The CBCT datasets were retrospectively obtained as part of routine clinical imaging. Therefore, the voxel size of 0.600 mm and the reconstructed slice thickness of 1 mm should be considered when interpreting measurements of relatively small anatomical structures, particularly mandibular canal diameter. Partial-volume effects and limited spatial resolution may have influenced the precision of small absolute measurements. In addition, the single-center retrospective design and the demographic differences observed among the study groups may limit the generalizability of the findings. Further multicenter prospective studies using higher-spatial-resolution imaging protocols and clinically correlated outcomes are warranted to validate these findings.

## 5. Conclusions

The findings of the present study demonstrate that, during SSRO planning, not only the presence of the mandibular canal but also the surrounding cortical and cancellous bone distances should be carefully evaluated. The presence of wider bony distances around the mandibular foramen and in the inferior region in retrognathic individuals may suggest a more favorable anatomical configuration in these regions. In contrast, the lower buccal cancellous bone thickness observed in prognathic individuals indicates that the inferior alveolar nerve may be positioned closer to the buccal cortex, potentially increasing the risk of neurosensory complications during the osteotomy, splitting, and fixation stages.

Clinically, detailed preoperative CBCT evaluation of the distances between the mandibular canal and the buccal, lingual, and inferior cortical structures should be performed, particularly in prognathic patients. The osteotomy line, screw placement, and split direction should be planned according to patient-specific mandibular morphology, and a more conservative surgical approach may be preferred in high-risk regions. In conclusion, consideration of mandibular sagittal position and the associated regional anatomical variations may contribute to more individualized preoperative assessment and surgical planning for SSRO.

## Figures and Tables

**Table 1:** Comparison of demographic characteristics of the patients according to the study groups, including sex distribution and mean age.

Variable	Group	Retrognathic	Normal	Prognathic	p1
		f (%)	f (%)	f (%)	
Sex	Female	36 (75) a	28 (58.3) a	16 (33.3) b	0.004
	Male	12 (25) a	20 (41.7) a	32 (66.7) b	
Variable		Mean ± SD	Mean ± SD	Mean ± SD	p2
Age		25.79 ± 4.87 A	31.21 ± 7.83 B	25.29 ± 4.12 A	0.000

p1: Significance value of the chi-square test; a, b: Results of the Bonferroni-corrected pairwise comparisons performed for significant chi-square test findings, where different lowercase letters indicate statistically significant differences between groups. p2: Significance value of the ANOVA test; A, B: Results of Tukey’s multiple comparison test performed following significant ANOVA findings, where different uppercase letters indicate statistically significant differences between groups.

**Table 2:** Comparison of the distance between the FBLC and the mandibular foramen, the distance between the buccal cortex and the mandibular canal, and mandibular canal diameter measurements in the mandibular nerve entrance region according to the study groups.

Measurement	Retrognathic ^1^	Normal ^2^	Prognathic ^3^	*p*	Difference
	Mean ± SD	Mean ± SD	Mean ± SD		
Distance between the FBLC and the mandibular foramen	7.83 ± 2.83	6.47 ± 2.51	7.05 ± 3.09	0.046	1 > 2
Distance between the buccal cortex and the mandibular canal	3.9 ± 1.26	3.22 ± 0.75	3.55 ± 1.36	0.018	1 > 2
Mandibular canal diameter	3.67 ± 0.87	3.5 ± 0.66	3.45 ± 0.87	0.368	

*p*: Significance value of the one-way ANOVA test; Tukey’s multiple comparison test was used to determine between-group differences. ^1^: Retrognathic, ^2^: Normal, ^3^: Prognathic. FBLC: The fusion between the buccal and lingual cortices.

**Table 3:** Comparison of the distance between the mandibular canal and the alveolar crest, buccal cortical bone thickness, buccal cancellous bone thickness, mandibular canal diameter, lingual cancellous bone thickness, lingual cortical bone thickness, and the distance between the mandibular canal and the mandibular base in the ramus–corpus transition region according to the study groups.

Measurement	Retrognathic ^1^	Normal ^2^	Prognathic ^3^	*p*	Difference
	Mean ± SD	Mean ± SD	Mean ± SD		
Distance between the mandibular canal and the alveolar crest	13.27 ± 3.36	12.29 ± 3.03	13.1 ± 3.36	0.294	
Buccal cortical bone thickness	2.8 ± 0.75	2.58 ± 0.47	2.51 ± 0.6	0.061	
Buccal cancellous bone thickness	2.97 ± 2.18	2.25 ± 1.27	2.33 ± 1.7	0.094	
Mandibular canal diameter	3.04 ± 0.65	3.08 ± 0.66	3.04 ± 0.61	0.95	
Lingual cancellous bone thickness	1.25 ± 1.26	1.08 ± 0.9	1.27 ± 1.02	0.616	
Lingual cortical bone thickness	2.25 ± 0.99	1.86 ± 0.46	1.83 ± 0.57	0.006	1 > 2.3
Distance between the mandibular canal and the mandibular base	8.2 ± 2.3	7.9 ± 1.94	7.77 ± 2.28	0.606	

*p*: Significance value of the one-way ANOVA test. For variables showing statistical significance, intergroup differences were evaluated using Tukey’s multiple comparison test. ^1^: Retrognathic, ^2^: Normal, ^3^: Prognathic.

**Table 4:** Comparison of the distance between the mandibular canal and the alveolar crest, buccal cortical bone thickness, buccal cancellous bone thickness, mandibular canal diameter, lingual cancellous bone thickness, lingual cortical bone thickness, inferior cancellous bone thickness, and inferior cortical bone thickness in the mesial region of the second molar according to the study groups.

Measurement	Retrognathic	Normal	Prognathic	*p*	Difference
	Mean ± SD	Mean ± SD	Mean ± SD		
Distance between the mandibular canal and the alveolar crest	14.99 ± 3.65	14.58 ± 2.56	15.66 ± 2.69	0.211	
Buccal cortical bone thickness	2.98 ± 0.79	2.93 ± 0.52	3.31 ± 0.67	0.035	3 > 1.2
Buccal cancellous bone thickness	3.28 ± 1.82	3.32 ± 1.49	3.02 ± 1.52	0.032	1.2 > 3
Mandibular canal diameter	2.91 ± 0.78	2.8 ± 0.48	2.85 ± 0.47	0.645	
Lingual cancellous bone thickness	1.03 ± 1.21	0.55 ± 0.53	0.77 ± 0.69	0.024	1 > 2
Lingual cortical bone thickness	1.84 ± 0.61	1.72 ± 0.54	2.1 ± 0.67	0.009	3 > 1.2
Inferior cancellous bone thickness	4.25 ± 2.25	3.05 ± 1.5	3.63 ± 2.15	0.015	1 > 2
Inferior cortical bone thickness	3.57 ± 0.77	3.6 ± 0.75	3.92 ± 0.94	0.026	3 > 1.2

*p*: Significance value of the one-way ANOVA test. For variables with statistically significant results, intergroup differences were evaluated using Tukey’s multiple comparison test.

## Data Availability

The raw data supporting the conclusions of this article will be made available by the authors on request.

## Abbreviations

The following abbreviations are used in this manuscript:

ALARA	As Low As Reasonably Achievable
ANB	Point A-nasion-Point B angle
ANOVA	Analysis of variance
CBCT	Cone-beam computed tomography
FOV	Field of view
IAN	Inferior alveolar nerve
ICC	Intraclass correlation coefficient
SD	Standard deviation
SNB	Sella-nasion-Point B angle
SPSS	Statistical Package for the Social Sciences
SSRO	Sagittal split ramus osteotomy
